# Sulfur-Containing Monoterpenoids as Potential Antithrombotic Drugs: Research in the Molecular Mechanism of Coagulation Activity Using Pinanyl Sulfoxide as an Example

**DOI:** 10.3389/fphar.2018.00116

**Published:** 2018-02-19

**Authors:** Liliya E. Nikitina, Sergei V. Kiselev, Valeriya A. Startseva, Andrei V. Bodrov, Zulfiya R. Azizova, Olga T. Shipina, Inna V. Fedyunina, Sergei V. Boichuk, Olga A. Lodochnikova, Vladimir V. Klochkov, Leisan F. Galiullina, Aliya V. Khaliullina

**Affiliations:** ^1^Department of General and Organic Chemistry, Kazan State Medical University, Kazan, Russia; ^2^Medical Physics Department, Institute of Physics, Kazan Federal University, Kazan, Russia; ^3^Department of Chemistry and Technology of Macromolecular Compounds, Kazan National Research Technological University, Kazan, Russia; ^4^A.E. Arbuzov Institute of Organic and Physical Chemistry, Kazan Scientific Center, Russian Academy of Sciences, Kazan, Russia

**Keywords:** terpenes, sulfoxides, platelets aggregation, coagulation activity, molecular mechanism of coagulation activity

## Abstract

In this article we present the synthesis of enantiomerically pure sulfoxide and study the influence of this compound on hemostasis. Detailed NMR studies and molecular dynamics simulations using sodium dodecyl sulfate (SDS) membrane models indicated that the bicyclic fragment of sulfoxide was embedded into the SDS micelle whereas the -SO(CH_2_)_2_OH fragment remained on the surface of the micelle and was in contact with the solvent. We also found that the pro-coagulative activity of sulfoxide was due to its ability to inhibit platelet activation and inhibited the catalytic activity of phospholipid surface which was involved in formation of coagulation clotting factor complexes.

## Introduction

It is well-known that ischemic heart disease and ischemic strokes are the world leaders among the cardiovascular diseases. The changes in the vascular wall caused by atherosclerosis promote the activation of platelets and coagulation hemostasis, which ultimately lead to the formation of a thrombus substantially reducing or completely blocking the blood flow. Affection of vascular wall, activation of platelets and coagulation hemostasis caused by atherosclerosis lead to formation of thrombus. Adhesion and aggregation of platelets promoted by the activation of plasmatic pro-coagulants on phospholipid cell surface contacting with blood cause thrombosis. Notably, cardiovascular diseases are connected with increased sensitivity of platelets to adhesion and aggregation to inductors. Activation of specific platelet receptors leads to a reorganization of their membrane, a disruption in the asymmetry of the location of phospholipids, and the appearance of mesomorphic structures (Finegold et al., 2013; Krudysz-Amblo et al., 2015).

Drugs inhibiting platelet activity are used to treat and prevent this group of diseases. Currently used drugs do not guarantee sufficient prevention and treatment of acute cardiovascular diseases. E.g., although the exact prevalence is unknown, up to 60% of patients taking acetylsalicylic acid might have different degrees of so-named “aspirin resistance” (Gasparyan et al., 2008). Therefore, the investigation of the substances that are capable to affect and normalize the processes indicated are the main objects of creating new antithrombotic drugs.

On the other hand, in some cases it is necessary to preserve the functional activity of platelets. Platelets concentrate is utilized to treat the patients with various types of thrombocytopenia. Clinical effectiveness of donor platelets depends on functional activity of platelets. The damage of vascular wall during the donor blood donation procedure and stress hormone adrenaline level increasing lead to platelet adhesion and activation, and result in appearing of the microvesicles. In addition, storage of platelets decreases their biological activity. Despite sodium citrate or heparin are commonly used as the anticoagulants of blood products preservation, the stabilizing effects of these compounds are insufficient due to formation of the platelets microvesicles. This fact also remains a driving force to find out the novel effective blood preserving agents.

It has been found that about 80% of people use traditional medicine for their primary health care. Most of these therapies involve the use of plant extracts or their active compounds, such as monoterpenoids. Recently pharmacological activity of some herbal substances has already been studied. Coagulation activities were confirmed for some of the phyto-compounds (Balick et al., 1994). It was also shown that monoterpenes might be the promising agents for prevention and treatment of cardiovascular diseases (Santos et al., 2011). On the other hand, sulfur is biogenic element and sulfur-containing compounds are widely represented both in natural products and synthetic biologically active substances. Combination of terpene fragment and biogenic sulfur-containing functional groups into one molecule allows to obtain new compounds with biologically active properties. The most available natural mono- and bicyclic monoterpenes such as camphene, α- and β-pinenes, limonene, carvone, 3-carene, and their derivatives—epoxides, epysulfides, alcohols, cyclopropenes, and allenes are usually used as initial compounds for our synthesis. We have recently created a series of sulfur-containing terpenoids of varied structures with the large set of the functional groups (Ishmuratov et al., 2014). Moreover, we also demonstrated the anti-fungal, anti-inflammatory, anti-helicobacter, antimicrobial and other types of activities of sulfur-containing monoterpenoids (Nikitina et al., 2009, 2010, 2012a,b; Gavrilov et al., 2010). Coagulation activity of these compounds has already been described in our previous publications (Kiselev et al., 2017; Nikitina et al., 2017a,b). We observed that all synthesized sulfur-containing monoterpenoids have a low toxicity, whereas mutagenic and genotoxic effects were not found (Nikitina et al., 2011).

Sulfoxides are known to be very attractive tools in organic synthesis as chiral auxiliaries or ligands for catalysis and in the pharmaceutical industry for their biological activity (Anderson et al., 2006; Alfonsov et al., 2008; Carreno et al., 2009; Wojaczynska and Wojaczynski, 2010).

**Scheme 1:**
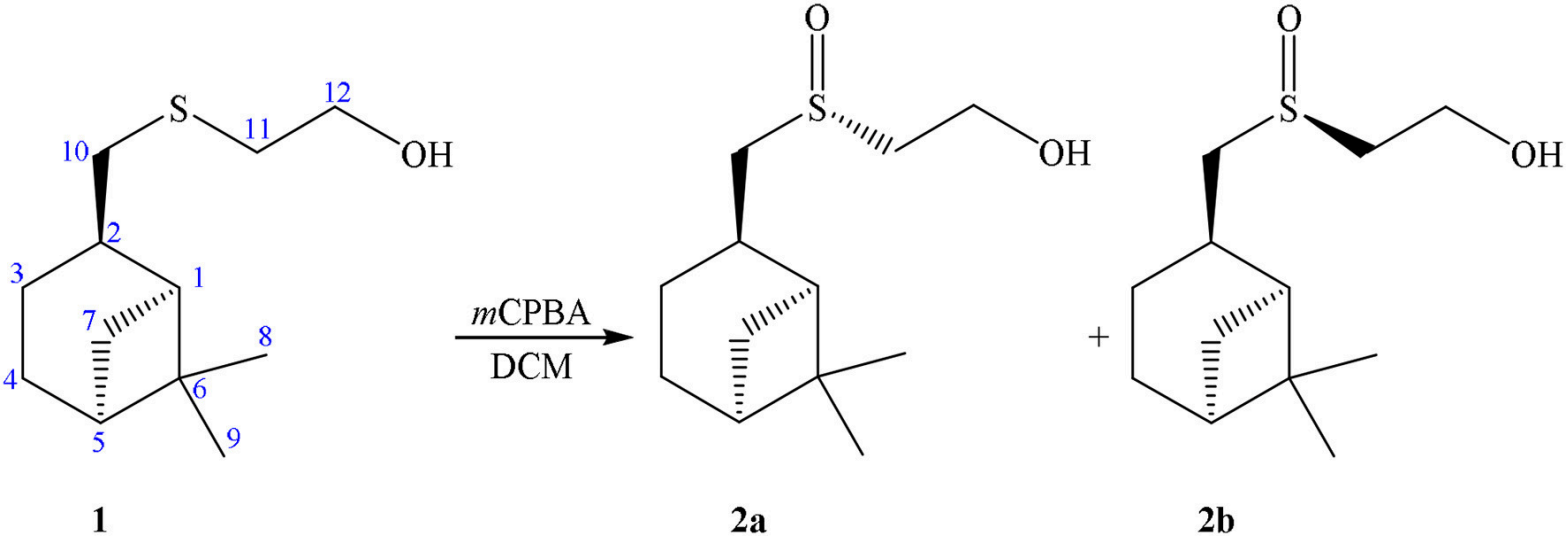
Synthesis of sulfoxide **2**.

Previously we have ascertained a stable “racemic compound-like” behavior of diastereomeric mixture of sulfoxides **2a**+**2b** obtained by the oxidation of enantio-pure sulfide derivative of (–)-β-pinene (Startseva et al., 2014). The oxidation of a β-hydroxysulfide **1** using *m-*chloroperbenzoic acid resulted in the formation of the corresponding β-hydroxysulfoxide **2** as a mixture of two diastereomers in 4:5 ratio (see Scheme 1).

Attempts to separate diastereomers **2a** and **2b** using column chromatography or fractional crystallization have failed. It turned out that the sample of **2** crystallized as an asymmetric dimer containing a supramolecular centro-symmetric moiety formed through S = O··H–O interactions between the present two independent molecules. The β-pinene skeleton keeps its natural configuration, while the configurations of the sulfur atoms are opposite. The sample of **2** is interesting as the first example of the co-crystallization of chiral sulfur compounds. Moreover, the dimer **2a**+**2b** can crystallize in two so-called “packing polymorphs” (Grant, 1999). The instances of polymorphism in a system with conserved hydrogen bonded synthons are rare (Fucke et al., 2010). In addition triclinic and monoclinic modifications of diastereomeric sulfoxides cocrystal, remarkable alterations in unit cell parameters by transition from 293 to 150 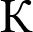 were ascertained (Lodochnikova et al., 2015; Nikitina et al., 2017c).

The importance of pure optical isomer forming in synthesis of potential biologically active compounds is widely accepted. In this article we present the synthesis of enantiopure sulfoxide **2a** and study the influence of this compound on hemostasis.

## Materials and methods

### Chemistry

Previously, we performed the addition of 2-mercaptoethanol to the double bond of (–)-β-pinene in the presence of ZnCl_2_ resulting in sulfide **1** with *cis*-configuration of the sulfide group relatively to the *gem*-dimethyl fragment of the molecule (Nikitina et al., 2006).

**2-((*S*)-(((1*S*,2*R*,5*S*)-6,6-dimethylbicyclo[3.1.1]heptan-2-yl)methyl)sulfinyl)ethan-1-ol (2a):** to a 0.0125 mmol of Ti(O*i*-Pr)_4_ (*R*)-mandelic acid (0.025 mmol) in 5 mL of CCl_4_ was added dropwise. After 1 h to a mixture 2 mmol of sulfide **1** was added, and through 0.5 h−4 mmol of *t*-BuOOH. The reaction mixture was stirred for 14–20 h to complete conversion of the starting sulfide (TLC control). Sulfoxide **2a** was isolated by column chromatography in silica gel and was purified by recrystallization from a mixture of acetone and CH_2_Cl_2_ (1:1). Sulfoxide **2a** is a white needle crystals {yield 80%, mp 98°C, [α]_D_^20^ = −54,50° (c = 1.00, acetone)}.

### Biochemical assays

Blood was obtained from healthy volunteers (average 26 ± 5-year-old) not taking aspirin, nonsteroidal antiinflammatory drugs, or other medications known to affect clotting factors or platelet function for at least 7–10 days, with informed consent from all subjects and approval by the Ethical Committee of Kazan State Medical University. All procedures were carried out in accordance with the approved guidelines. All subjects gave the written informed consent in accordance with the Declaration of Helsinki.

Cytotoxicity of a sulfoxide **2a** was examined by the MTS (3-(4,5-dimethylthiazol-2-yl)-5-(3-carboxymethoxyphenyl)-2-(4-sulfophenyl)-2*H*-tetrazolium)-based assay (Niks and Otto, 1990) with minor modifications. Briefly, human BJ fibroblasts (ATCC, USA) were seeded in 96-well flat-bottomed plates (Corning Inc., Corning NY) and allowed to attach and grow for 24 h in complete medium DMEM/199 supplemented with 10% of fetal veal serum (FBS, GIBCO, GrandIsland, NY), L-glutamine (0,3 mg/ml) and antibiotics penicillin-streptomycin (Paneko, Russia), The cells were cultured at 37°C of 5% of CO_2_ (LamSystems, Russia) with the indicated concentrations of pinanylsulfoxide 2a, 1.5% solution of ethyl alcohol or chemotherapeutic agent etoposide (Calbiochem, USA) (control). Finally, 3-(4,5-dimethylthiazol-2-yl)-5-(3-carboxymethoxyphenyl)-2-(4-sulphophenyl)-2*H*-tetrazolium, inner salt (MTS) reagent (Promega, Madison, WI) was added to the culture medium to assess the live cell numbers. The cells were incubated with MTS reagent for at least 1 h and assayed at 492 nm on MultiScan FC plate reader (Thermo Fisher Scientific, Waltham, MA). The data was normalized to the control group.

The ability of sulfoxide **2a** to correct hemostasis *in vitro* was also examined. For this, the venous blood was obtained by cubital vein puncture and stabilized with sodium citrate solution 3.8%. Blood was centrifuged (10 min, 1,000 rpm) for platelet-rich plasma preparation. The upper layer of plasma was then transported into another tube and the remaining portion of the blood was centrifuged (20 min, 3,000 rpm) to obtain platelet-poor plasma which was then used for dilution of platelets-rich plasma up to fixed volume concentration of platelets and determination of coagulating hemostasis. The anticoagulant activity of sulfoxide **2a** was measured by activated partial thrombosis time (aPTT) and prothrombin time (PT). Aggregating activity of platelets were determined by analyzer “Chrono-Log Corporation” (USA) using the method of Born (1962). For this purpose, plasma received from the venous blood of patients with ischemic heart disease (IHD) and patients with evident changes in the hemostasis system was used. The induced platelets aggregation was studied on plasma obtained from healthy donors [City Clinical Hospital N 7, Center of Emergency Medicine (Kazan)]. 0.05 ml solution of 10% of ethyl alcohol containing from 0.125 to 8 mM of sulfoxide was added to 0.45 ml platelet rich plasma, and this mixture was incubated for 5 min at a temperature of 37°C. In control experiments the solvent (10% solution of ethyl alcohol used for preparation of the compound) was added to the plasma. Coagulant activity was determined using “Automatic hemostasis analyzer” (ACL TOP 500 Instrumentation). Solutions of ADP (adenosine diphosphate) (5 μM), adrenaline (10 μM), collagen (2 μg/mL), arachidonic acid (0.5 mM) and ristomycin (1 mg/mL) were applied as inductors of platelets aggregation. The same volume of plasma without platelets was taken as the optical control. The aggregation degree was evaluated by the maximum incidence value of the optical density after the reaction compared with the original value. Relative efficiency of obtained compound was determined by comparison with acetylsalicylic acid. For this purpose the plasma of patients with IHD taking acetylsalicylic acid was used. Platelet concentrate was obtained from blood of healthy donors stabilized by sodium citrate by automatic cytopheresis on the device “Haemonetics Corporation MSC+, USA.” Cytopheresis of platelets was performed using the principle of intermittent flow through a separating chamber. Platelet concentrate was stored in bags made of special plastic for the platelets collection (“MSC Haemonetics corporation+,” USA) for 5 days at a temperature of 22–24°C and constant stirring by platelet mixer (“Presvak,” Argentina). Platelet concentrate was stabilized by ACD-A (anticoagulant citrate dextrose solution) at a ratio of 9:1 containing 8 g of citric acid monohydrate, 22 g sodium citrate, 24.5 g of glucose monohydrate and water up to 1,000 ml. Samples from platelet concentrate were taken in test tubes type of “Vacutainer,” and the number of microvesicles was determined on a flow cytometer BD FACScanto RUO (Becton Dickinson, USA) after dilution with phosphate buffer (Becton Dickinson, USA). The absolute number of microvesicles in 1 mm was counted by light diffusion for a fixed time (60 s) using the CellQuest program (Iversen et al., 2013). Thrombogenic properties of microvesicles were determined by thrombodynamics evolution and surface-dependent standard coagulation tests: aPTT and prothrombin time. Samples of platelet concentrate were centrifuged for 20 min at 2,500 g, and 0.1 ml of supernatant fluid was added to 0.9 ml of plasma get from healthy donors. Thrombodynamics of plasma was estimated by fibrin formation rate on device “Thrombodynamics recorder T-2” (Russia) using video recording of the growth of fibrin clot in the space with coagulation activation from the surface with immobilized tissue factor.

### Statistic analysis

All procedures were performed by using Graph Pad Prism 6. The results were analyzed with Kolmogorov-Smirnov test and Kruskal-Wallis test. The results were presented as average values and standard deviations (σ). A comparative study was performed applying the criterion of pair *t*-test. The differences were considered to be significant at *p* < 0.05.

### NMR spectroscopy

All NMR (nuclear magnetic resonance) experiments were performed on a Bruker Avance II-500 NMR spectrometer [500 MHz (^1^H)] equipped with a 5 mm probe using standard Bruker TOPSPIN software at *T* = 293 K. ^1^H NMR spectra were recorded using 90° pulses with duration of 7.0 μs, delay between pulses of 2 s, a spectrum width of 12 ppm and a minimum of eight scans. Complete assignment of the ^1^H NMR spectrum of the title compound was accomplished by 2D ^1^H-^1^H COSY, ^1^H-^13^C HSQC and ^1^H-^13^C HMBC NMR experiments. Chemical shifts were given in values of ppm, referenced to a residual solvent signal. The samples were prepared by dissolving in D_2_O with a concentration of 9.4 mM. The solution volume was 0.6 mL. Micelles of sodium dodecylsulfate (SDS) were obtained by dissolving SDS in D_2_O to a final concentration of 40 mM (Caillon et al., 2013). 2D NOESY experiments were performed with pulsed filtered gradient techniques. The relaxation delay was set to 2 s and the 90° pulse length to 7.5 μs. Mixing time value in 2D NOESY experiment was 0.2 s.

### Molecular dynamics simulations

All MD (molecular dynamics) simulations were performed with the GROMACS 5.01 package (Abraham et al., 2014) using GROMOS53A6 force field. Initial coordinates of the SDS micelle, which contains 60 dodecylsulfate molecules, were obtained from simulations carried out by A. MacKerell (MacKerell, 1995). The starting coordinates of sulfoxide **2a** molecule and topology based on the GROMOS96 53a6 force field for the sulfoxide **2a** as well as for the SDS molecule were obtained from Automated force field Topology Builder (ATB) (Malde et al., 2011). Charges to the atoms in the headgroup of SDS were assigned as defined in Bruce et al. (2002). The micelle was initially centered in a cubic box with a periodic image distance of 6.46 nm. Sulfoxide **2a** was placed at a distance ~7 Å from the the SDS–water interface. The system was then solvated with ~8,000 SPC water molecules and 60 Na+ ions to keep the system electrically neutral (Berendsen et al., 1981). The sulfoxide **2a** was then positionally constrained and subjected to steepest descent minimization to reduce close contacts. Minimization was followed by heating to 300 K over 50-ps of NVT-MD run simulations. Then, subsequent isothermal isobaric ensemble (*P* = 1 bar, *T* = 300 K) simulation (NPT-MD) was performed for 3 ns to allow the micelle and water molecules to equilibrate. Final MD simulation was carried out for 20 ns. MD simulations were visualized and images were generated using the visual molecular dynamics (VMD) package (Humphrey et al., 1996).

### Single-crystal X-ray

The X-ray diffraction data for the crystals of sulfoxide **2a** (excluding structure factors) reported in this paper has been deposited with the Cambridge Crystallographic Data Centre as supplementary publication number CCDC 923760.

## Results

According to results of previous researches, optically active sulfoxides of the terpene series were prepared by the oxidation of the corresponding sulfides using various oxidizing agents, including *m-*chloroperbenzoic acid (Binns et al., 1985; Annunziata et al., 1987; Eschler et al., 1988; Pyne et al., 1989; Arai et al., 1991), sodium periodate (Vargas-Díaz et al., 2005), air oxygen (Nikitina et al., 2001), as well as by the Kagan-Modena and Bolm asymmetric oxidation methods (Yang et al., 1994; Aversa et al., 2002; Demakova et al., 2012a,b). We also reported that the crystal of the diastereomer **2a** might be obtained as a result of the crystallization after a careful separation of the dimer crystals (Startseva et al., 2014).

**Scheme 2:**
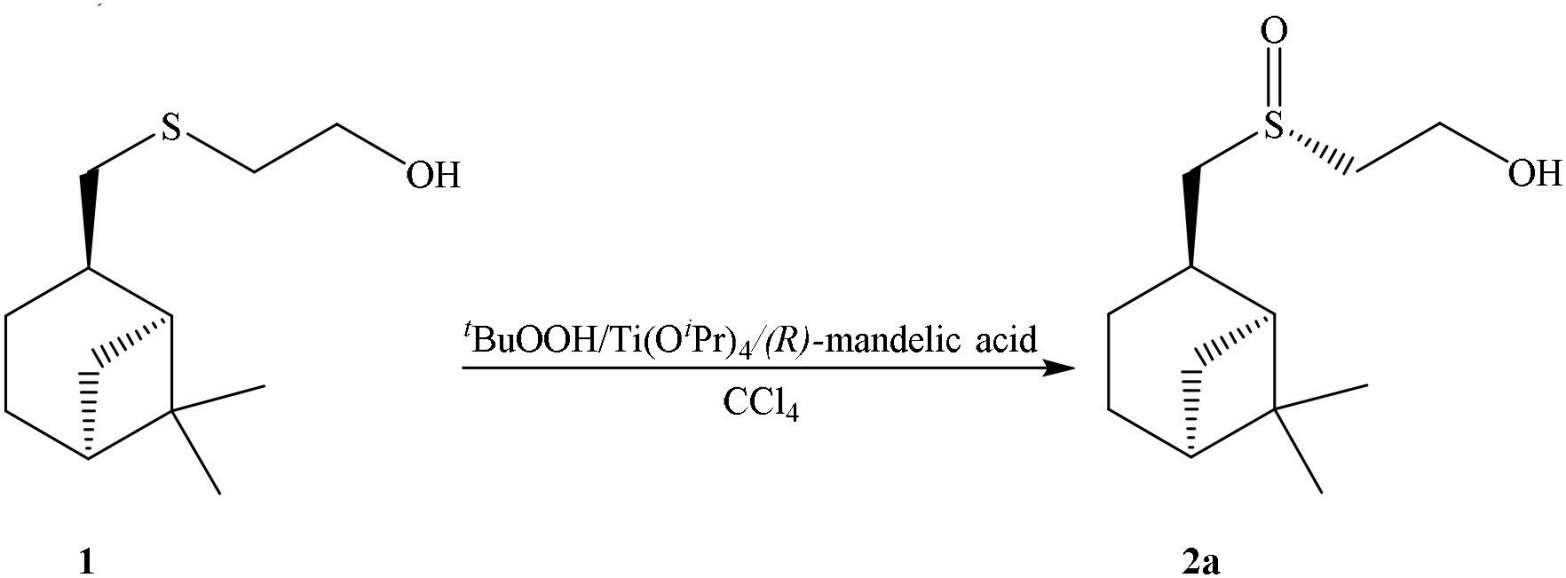
Synthesis of sulfoxide **2a**.

In our case sulfoxide **2a** was prepared by oxidizing of sulfide **1** according to Uemura method, using Ti(O*i*-Pr)_4_, (*R*)-C_6_H_5_CH(OH)COOH/*t*-BuOOH as the oxidation system (Scheme 2) (Komatsu et al., 1993).

**Figure 1:**
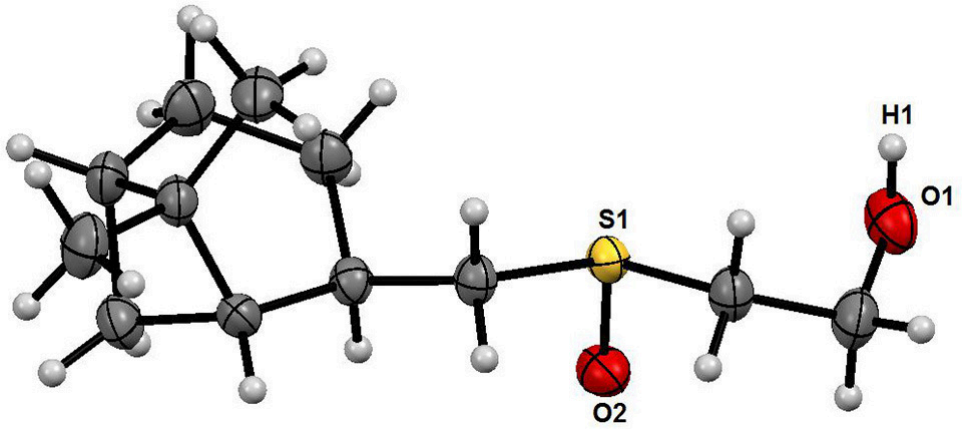
X-ray data of sulfoxide **2a**.

The structure of the sulfoxide **2a** was determined by X-ray structural analysis at room temperature. According to X-ray structural data, sulfoxide **2a** was presented as a single diastereomer form with the *S*-configuration of the sulfinyl group located relatively to the pinane framework of the molecule (Figure 1).

According to the NMR spectroscopy data the enantiomeric excess is close to 100%. Some of sulfoxide molecules would be in the form of dimer in the case of diastereomer formation, and that fact confidently proves the absence of second diastereomer. This, in turn, would have influenced the ^13^C NMR spectrum, in which two sets of signals would have been observed (Anderson et al., 2006), whereas there is only one set in the spectrum in this case (Table 1).

**Table 1:** ^1^H NMR chemical shifts (δ, ppm) and spin-spin interaction constants (in parenthesis *J*, Hz) of the compound in D_2_O and D_2_O+SDS solutions at 293 K^*^.

**Atoms**	**in D**_**2**_**O solution**		**in D**_**2**_**O**+**SDS solution**	
	**δ ^1^H, ppm (J, Hz)**	**δ ^13^C, ppm**	**δ ^1^H, ppm**	**δ ^13^C, ppm**
CH-1	1.81 ov	46.04	1.87 ov	46.02
CH-2	2.45 m	34.72	2.48 m	34.79
CH_2_-3	1.54; 1.99 m	20.27	1.52; 2.03 m, ov	20.51
CH_2_-4	1.76; 1.86 ov	25.20	1.84; 1.90 ov	25.63
CH-5	1.81 ov	40.51	1.83 ov	40.60
CH_2_-6	0.89; 2.27 m	32.28	0.95; 2.30 m, ov	32.49
C-7	–	37.91	–	38.30
CH_3_-8	1.07 s	26.94	1.11 s	27.20
CH_3_-9	0.88 s	22.34	0.93 s	22.76
CH_2_-10	2.88; 2.98 ov	53.90	2.89; 2.95 ov	54.21
CH_2_-11	2.85; 2.95 ov	59.52	2.86 ov	59.84
CH_2_-12	3.87 (9.0; 3.9) q	54.59	3.90 ov	54.81

* *s, singlet; q, quadruplet; br, broadened signal; m, multiplet; ov, overlapped signal*.

**Figure 2:**
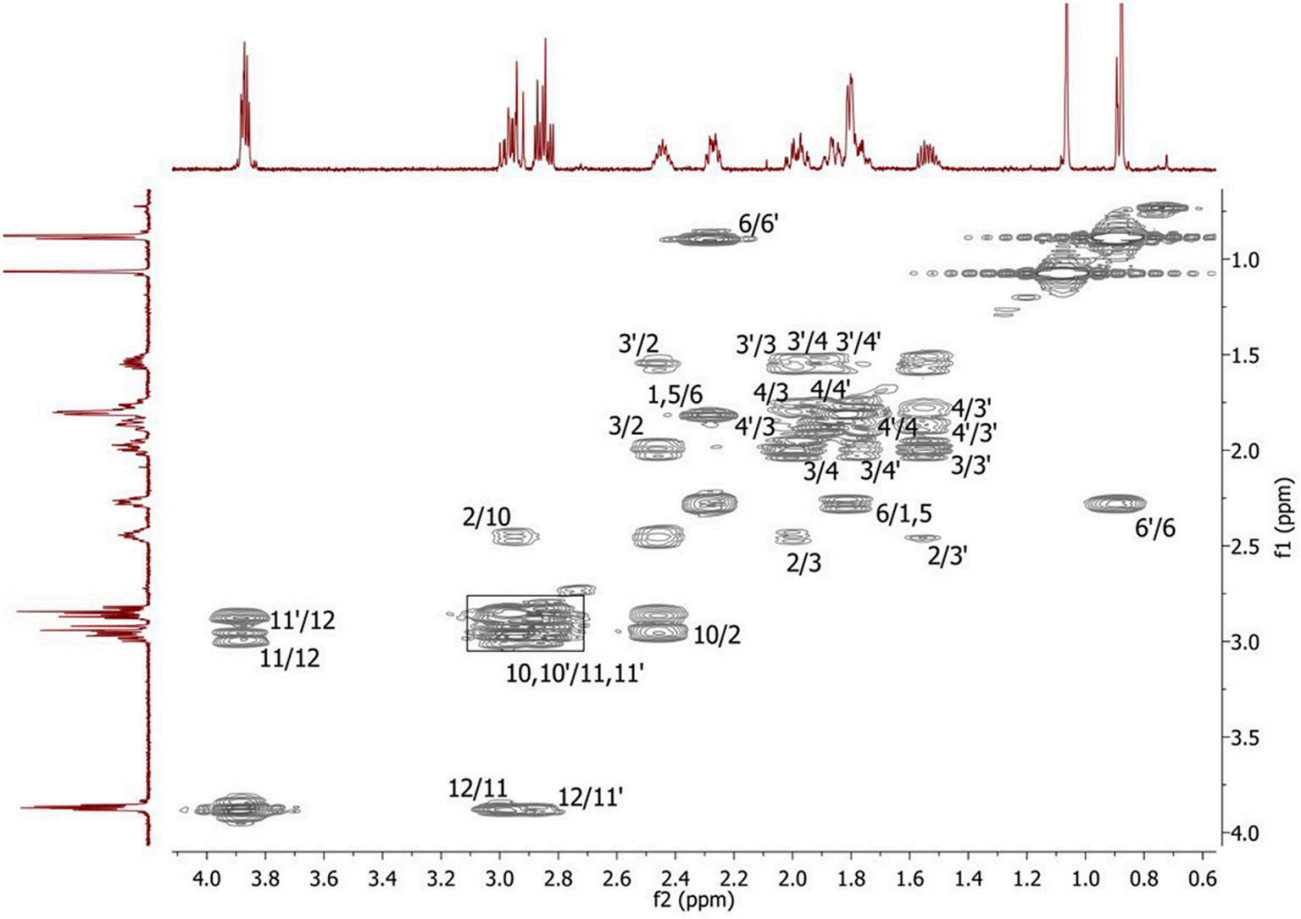
2D ^1^H-^1^H COSY spectrum of the compound in D_2_O, *T* = 293 K. The numeration of the protons corresponds to the Figure 1.

**Figure 3:**
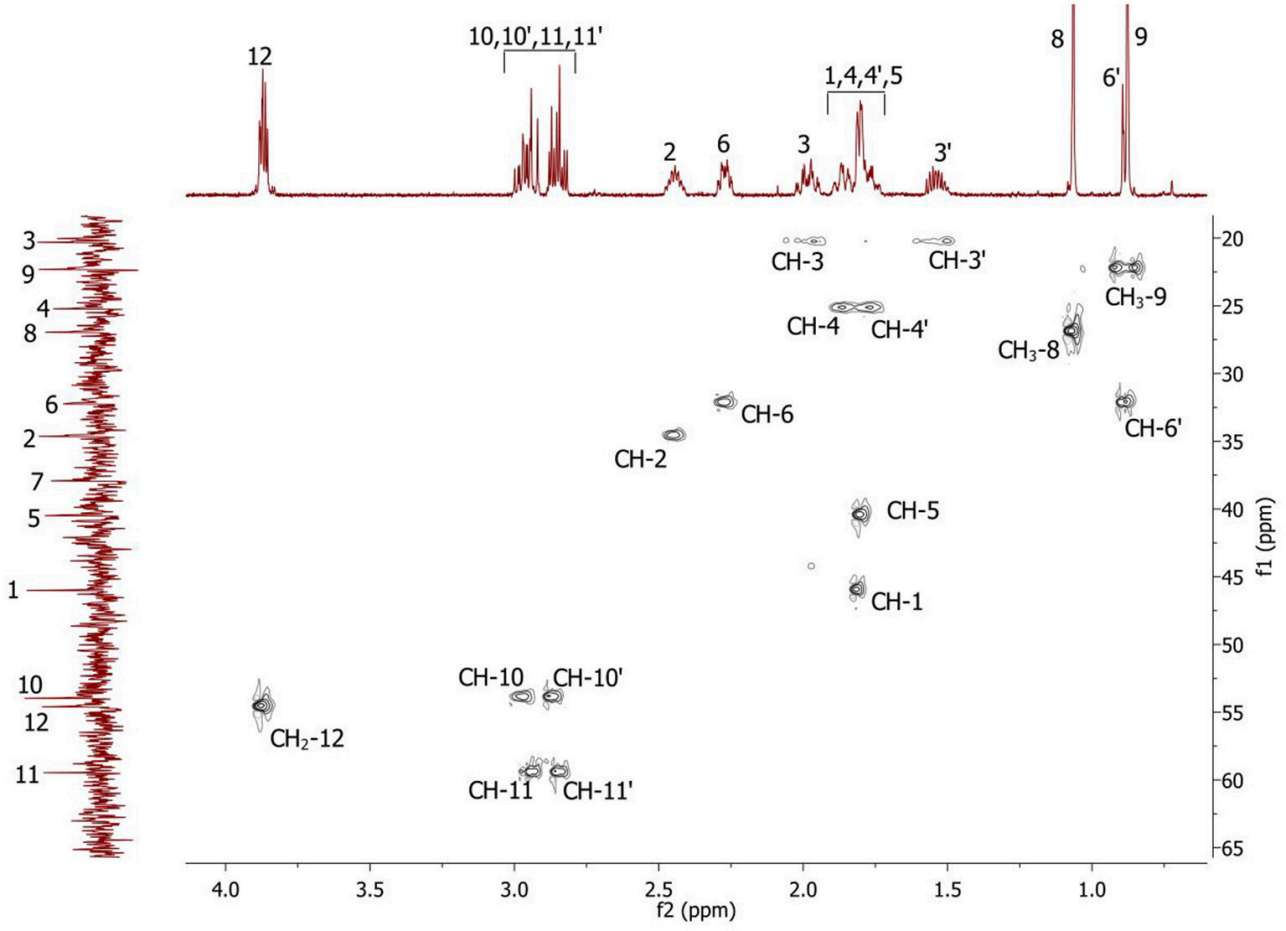
2D ^1^H-^13^C HSQC spectrum of the compound in D_2_O, *T* = 293 K. The numeration of the protons corresponds to the Figure 1.

**Figure 4:**
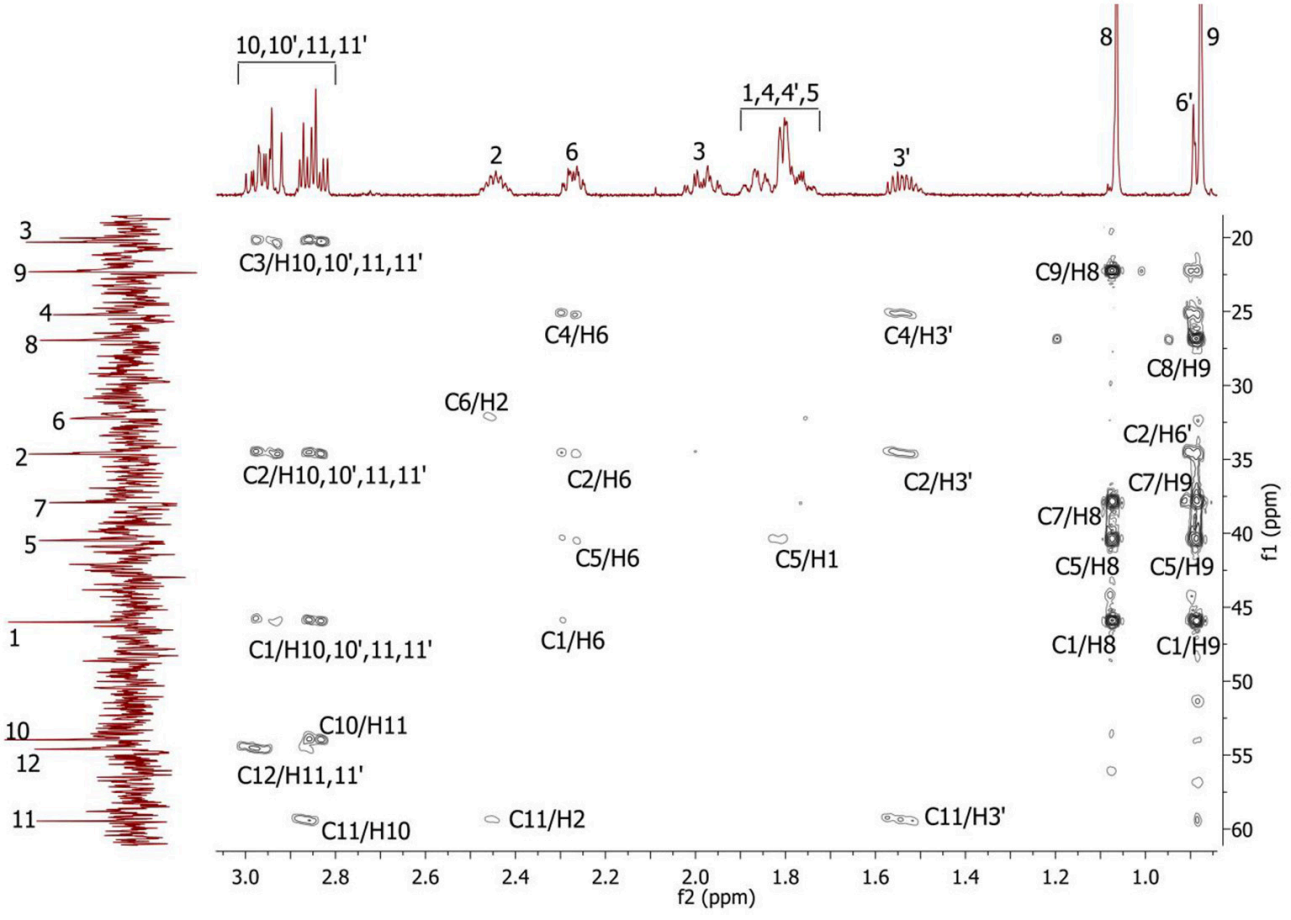
2D ^1^H-^13^C HMBC spectrum of the compound in D_2_O, *T* = 293 K. The numeration of the protons corresponds to the Figure 1.

Complete assignment of the ^1^H NMR spectrum of sulfoxide **2a** was accomplished by 2D ^1^H-^1^H COSY, ^1^H-^13^C HSQC and^1^H-^13^C HMBC NMR experiments (Figures 2–4) and based on the analysis of the signal multiplicities, the integral values and the characteristic chemical shifts. CH_3_-8 and CH_3_-9 protons are observed in the spectrum as two singlets with chemical shifts of 1.07 and 0.88 ppm respectively (Figure 7A). Geminal protons of CH_2_-12 resonate in the spectrum as AB-quadruplet at 3.87 ppm. According to ^1^H-^1^H COSY (Figure 2) and ^1^H-^13^C HSQC (Figure 3) correlations all protons signals and directly bonded to them carbon signals were assigned. It was established that geminal protons H6 and H6′ related to CH_2_-6 were highly non-equivalent. They were observed in the ^1^H NMR spectrum as two multiplets with chemical shifts 2.27 and 0.89 ppm. A similar pattern was observed for geminal H3 and H3′ protons related to CH_2_-3 (δ 1.99 and 1.54 ppm). CH_2_-4 protons signals overlapped with CH-1 and CH-5 resonance lines, but from 2D spectra it was established that this methylene group signals had chemical shifts 1.86 and 1.76 ppm. Geminal protons CH_2_-10 and CH_2_-11 were also pairwise non-equivalent. Corresponding multiplets were overlapped and had resonances in the ^1^H NMR spectrum in the area of 2.8…3.0 ppm. CH-1 and CH-5 chemical shifts were also defined according to 2D NMR experiments. They were observed in the spectrum as overlapped multiplets at 1.81 ppm. All bonded to protons carbon signals were assigned according to ^1^H-^13^C HSQC (Figure 3) spectrum. Finally, chemical shift of the C-7 signal was defined based on the results of ^1^H-^13^C HMBC experiment (Figure 4). All assignment of ^1^H and ^13^C NMR signals was also confirmed by ^1^H-^13^C HMBC correlations. Chemical shifts of all ^1^H and ^13^C NMR signals are shown in the Table 1.

**Figure 5:**
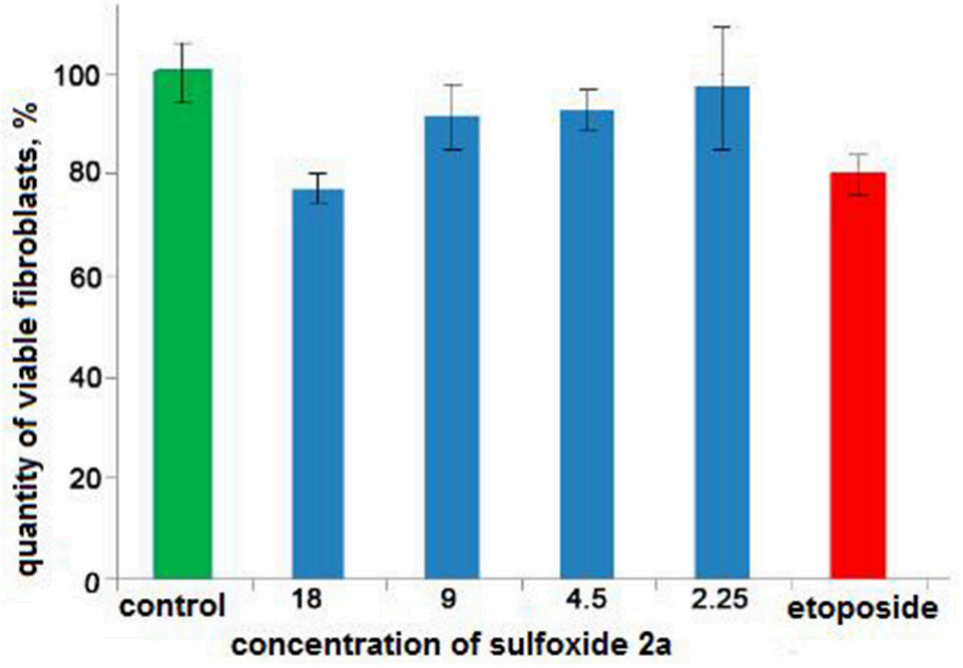
The results of the MTS-based assay of cytotoxicity of sulfoxide **2a** in the concentration range from 18 to 2.25 mM. Control is presented by 1.5% solution of ethyl alcohol. Cytostatic compound of comparison is etoposide (1.25 mM, Calbiochem, USA).

Recent studies have shown that sulfur-containing monoterpenoids have membranotropic properties (Kiselev et al., 2017). Therefore, in present study we examined the possibility of using sulfoxide **2a** as a platelet concentrate stabilizer. Two mmol concentration of sulfoxide **2a** was used as the most effective minimal concentration with anti-aggregative activity and low cytotoxicity (in this concentration ~100%-viability of the BJ fibroblasts was observed) (Table 2, Figure 5). Sulfoxide **2a** almost completely inhibited platelet aggregation induced by arachidonic acid, collagen and adrenaline in comparison with acetylsalicylic acid that decreased only ADP-induced aggregation in this concentration.

**Table 2:** Influence of sulfoxide **2a** on aggregation of platelets and indicators of coagulation hemostasis *in vitro* in patients with IHD.

**Inductor**	**Normal values, %**	**Control–donor's plasma, *n* = 5**	**Concentration of sulfoxide 2a, mM**							ACA^**^, mM
			**8**	**4**	**2**	**1**	**0.5**	**0.25**	**0.125**	**2**
Collagen, %	50–75	72 ± 3	0^*^	12 ± 6^*^	8 ± 4^*^	71 ± 3	72 ± 2	70 ± 3	68 ± 4	70 ± 2
Arachidonic acid, %	62–69	68 ± 2	0^*^	7 ± 4^*^	5 ± 2^*^	10 ± 4^*^	64 ± 2	70 ± 4	68 ± 2	62 ± 4
Adrenalin, %	60–71	67 ± 5	10 ± 2^*^	5 ± 2^*^	10 ± 2^*^	68 ± 4	73 ± 2	66 ± 4	68 ± 2	72 ± 2
ADP, %	50–75	66 ± 4	55 ± 2^*^	58 ± 3^*^	68 ± 2	66 ± 3	68 ± 2	64 ± 4	66 ± 2	47 ± 3^*^
Ristomycin, %	50–75	70 ± 3	68 ± 4	70 ± 2	66 ± 5	70 ± 2	68 ± 3	70 ± 2	68 ± 4	70 ± 2

n, number of studies;

* p < 0.001, statistically significant differences of samples compared with control;

** *Acetylsalicylic acid*.

**Figure 6:**
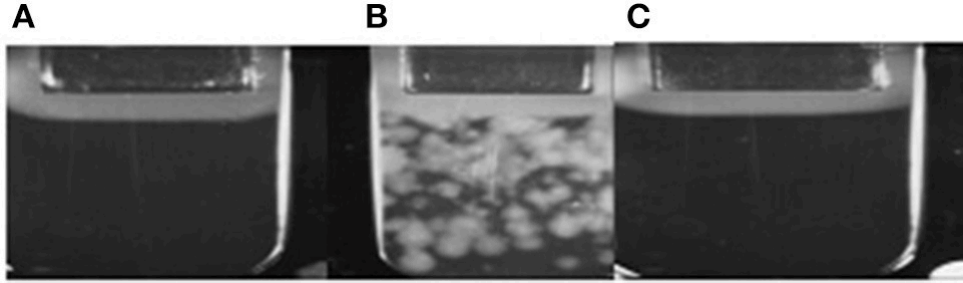
**(A)** Plasma obtained from healthy donors; **(B)** Plasma after addition of platelets sample; **(C)** Plasma after addition of platelets sample containing sulfoxide **2a**.

According to results of flow cytometry analysis, we observed almost 2.5-fold decrease in the numbers of microvesicles in platelet concentrates supplemented with sulfoxide **2a** when compared to the control samples. Important the numbers of microvesicles increased ~11-fold by the end of the storage period when compared to the initial time-point (Table 2). The microvesicles indicated above have the thrombogenic properties which were proved by activation of blood coagulation after 48 h of storage with adding 0.1 ml of supernatant from platelet concentrate to 0.9 ml of plasma from healthy donors. Hypercoagulation was confirmed by decreasing of aPTT and prothrombin time, significant increase of fibrin formation rate (V, Vi, Vst), fibrin clot size (Cs) and density (D), appearance of spontaneous clots (Tsp) in the test of thrombodynamics. Of note, all the parameters indicated above approached to the parameters of the control plasma in the presence of sulfoxide **2a**: aPTT and prothrombin time increased, and the rates of fibrin formation decreased. Removal of microvesicles from the donor plasma leaded to similar results, but fibrin clot formation rate decreased in more significant extent (Figure 6, Table 3).

**Table 3:** Influence of platelet concentrate containing sulfoxide **2a** on coagulation properties of plasma.

**Coagulation values**	**Normal values**	**Control, *n* = 5**	**Platelet concentrate**	**Platelet concentrate with sulfoxide**	**Plasma without microvesicules**
APTT, s	24–35	28.6 ± 1.2	22.5 ± 1.5^*^	31.4 ± 2.7^**^	30.6 ± 1.8
Prothrombin time, s	11–16	10.7 ± 0.8	8.9 ± 0.1^*^	12.1 ± 0.81^**^	11.9 ± 0.6
V—Fibrin clot growth retardation rate, μm/min	20–29	26.3 ± 2.2	39.4 ± 4.1^*^	31.7 ± 2.1^**^	20.0 ± 3.21^*^
Tlag—Fibrin clot retardation time, min	0.6–1.5	1.4 ± 0.1	1.39 ± 0.07	1.2 ± 0.12^**^	1.82 ± 0.08^*^
Vi—Fibrin clot growth initial rate, μm/min	39–51	49.5 ± 1.8	54.3 ± 0.2^*^	50.9 ± 0.8^**^	45.8 ± 0.8^*^
Vst—Growth stationary rate, min^−1^	22–28	26.3 ± 2.2	39.4 ± 4.1^*^	31.7 ± 2.1^**^	20.0 ± 3.2^*^
CS—size, μm	800–1,200	1154 ± 125	1443 ± 153^*^	1132 ± 32.1^**^	891 ± 53^*^
D—fibrin clot density, relative units	15,000–32,000	24981 ± 243	30818 ± 967^*^	2577 ± 228^**^	26121 ± 467^*^
Tsp—clot formation time, min	absent	absent	26.6 ± 0.3^*^	absent^**^	absent

n, number of studies;

* p < 0.05, statistically significant differences of blood test results compared to control plasma;

** *p < 0.05, statistically significant differences of blood results compared to plasma after platelet concentrate addition*.

In order to ascertain the possible interaction between sulfoxide **2a** and cell membranes, we utilized NMR spectroscopy. There are some difficulties in studying of interaction of sulfoxide with model phospholipid cell membrane by NMR. Proton transverse relaxation time of phospholipid aggregates is too short relative to the NMR chemical shifts time-scale and leads to significant broadening of the signals in the spectra. For this reason, sodium dodecyl sulfate micelles were used as model of cell membrane, which are extensively applied in similar NMR studies (Henry and Sykes, 1994; Lubecka et al., 2010; Galiullina et al., 2012, 2015; Usachev et al., 2013; Rakhmatullin et al., 2016). Head polar groups of SDS can be used to physically mimic a surface of biological membrane.

**Figure 7:**
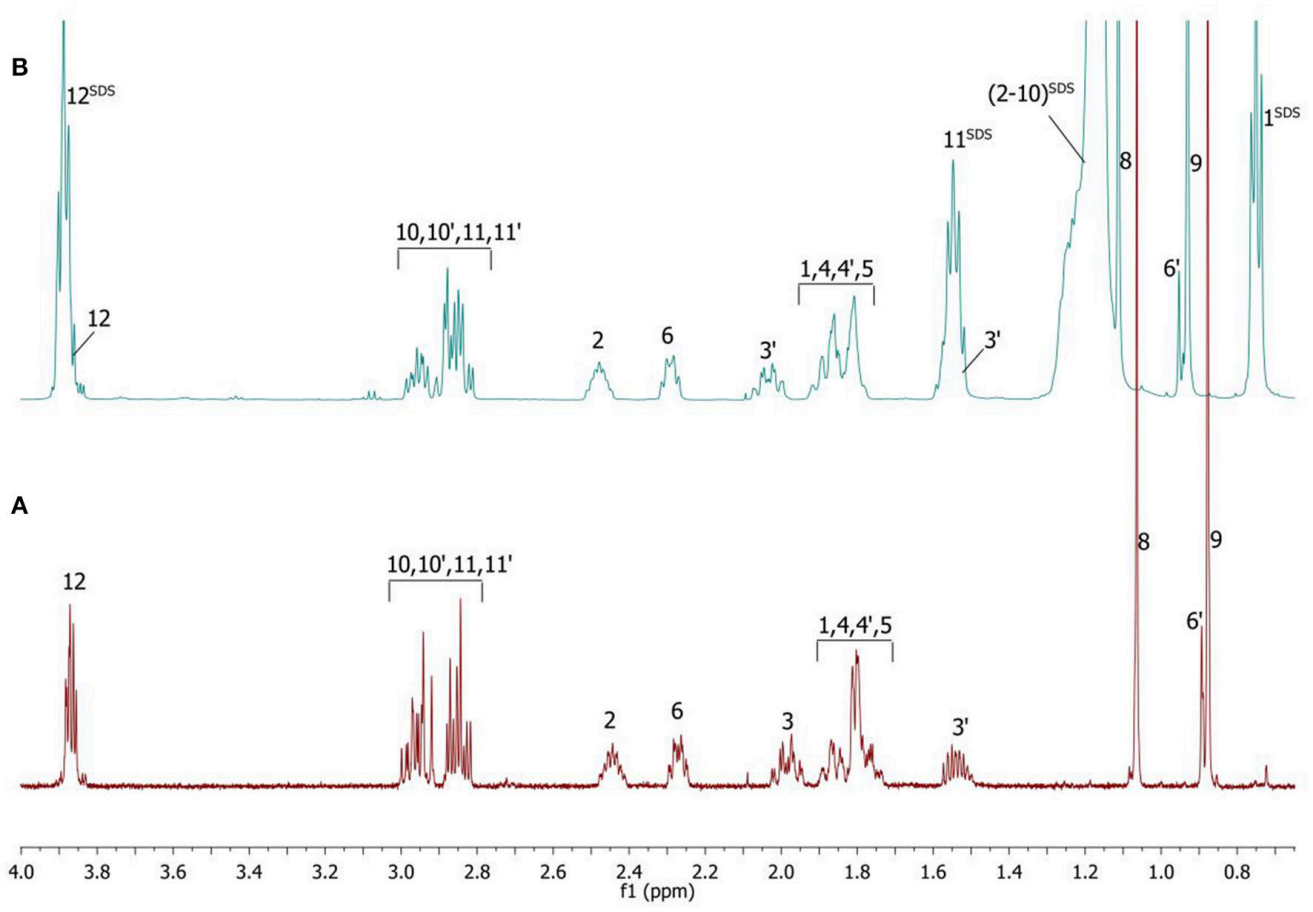
^1^H NMR spectra of the compound in D_2_O **(A)** and D_2_O+SDS **(B)** solutions, *T* = 293 K. SDS signals marked by superscript.

^1^H NMR spectrum of the compound is significantly changed after addition of SDS micelles (Figure 7B).

The signals are broadened and some of them are shifted if compared with the spectrum of the compound in pure D_2_O (see Table 1). Noticeable changes are observed for the signals CH_2_-10 and CH_2_-11. The signals are broadened and a number of lines in multiplet are decreased. H-11 and H-11′ geminal protons are become almost equivalent. Unfortunately, line shape changes of the signal CH_2_-12 cannot be analyzed because it is overlapped with SDS signal CH_2_-12^SDS^. However, the resonances of H-2, H-3, H-6,6′ and CH_3_-8,9 are shifted to lower fields. These differences between ^1^H NMR spectra of the compound in pure D_2_O and in D_2_O+ SDS micelles can be explained by interaction of terpene with model membrane. Dramatic changes of the signals CH_2_-10 and CH_2_-11 allow assuming that sulfoxide group of the terpene is responsible for binding of the compound with SDS micelle.

**Figure 8:**
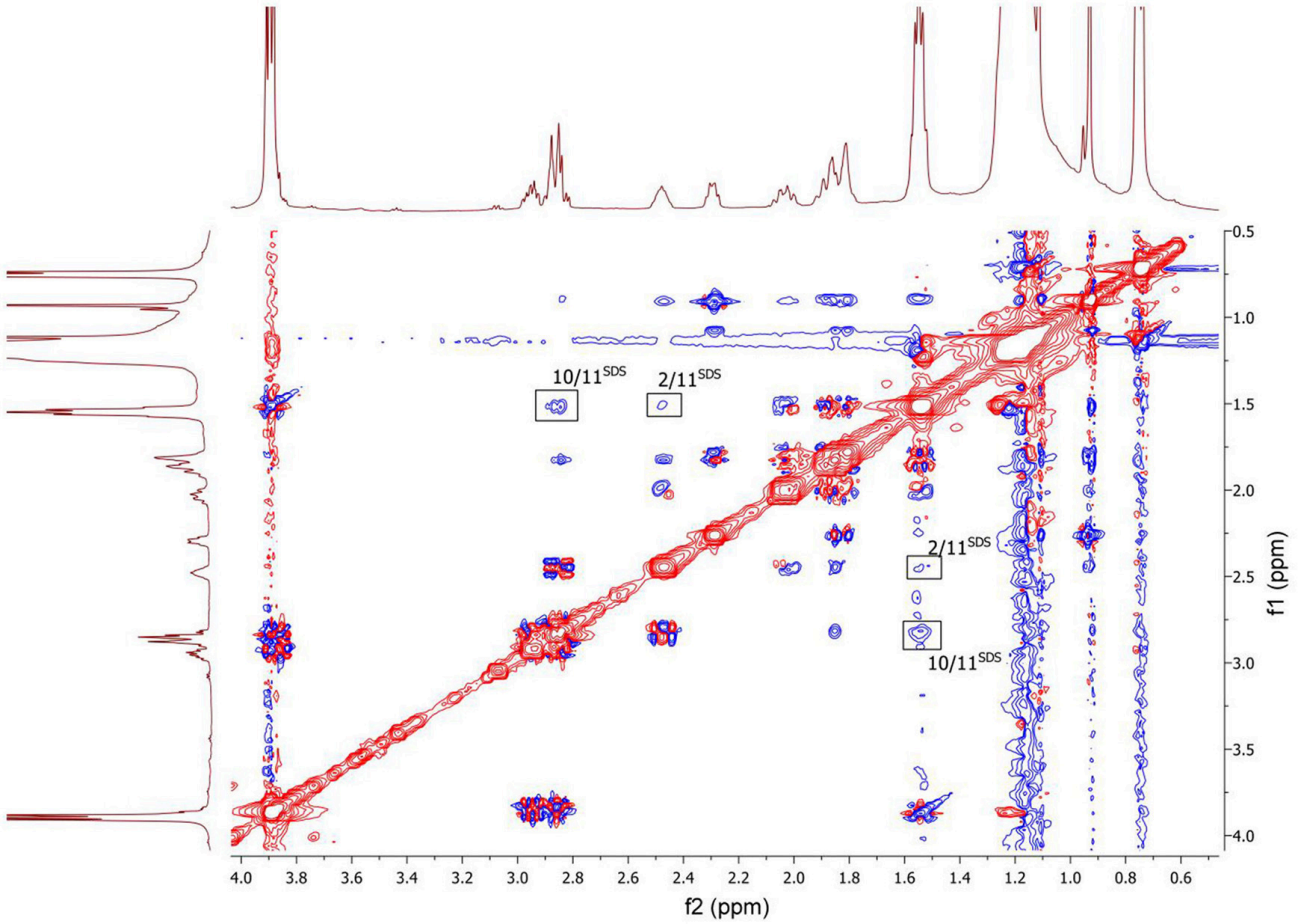
2D NOESY NMR spectrum of the compound in D_2_O+SDS solution, *T* = 293 K. Intermolecular NOEs are shown in bars. Mixing time was 0.2 s.

To clarify the mechanism of the complex formation between the terpene and SDS micelle 2D NOESY experiment was carried out (Figure 8). Several non-trivial intermolecular nuclear Overhauser effects (NOE) indicating close spatial location of the corresponding chemical groups of the terpene and SDS micelle were observed in the spectrum. The cross peaks between the signal CH_2_-11 of SDS (11^SDS^) and the signals CH-2 and CH_2_-10 of the compound showed that the compound slightly penetrated into the surface of the micelle. Probably, polar group S = O is a binding site of the compound in this complex.

**Figure 9:**
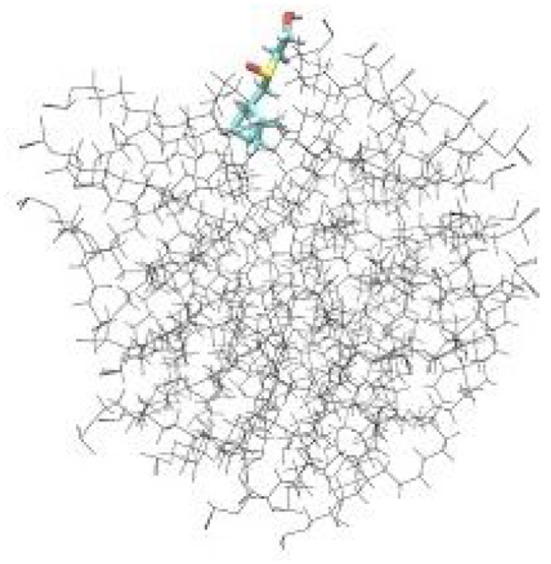
Results of molecular dynamics simulations of sulfoxide **2a** in SDS micelles.

To examine the detailed atomistic picture of sulfoxide **2a**/SDS micelle interactions, classical molecular dynamics simulations were performed. Figure 9 shows the snapshot of the system after 20-ns MD simulation. Visual examination of the sulfoxide **2a**/micelle complex indicates that sulfoxide is embedded with its bicyclic fragment inside the SDS micelle, whereas the -SO(CH_2_)_2_OH fragment of sulfoxide is located on the outer part of SDS micelle and is in contact with solvent.

## Discussion

Our present data confirms the anti-coagulation properties of sulfoxide **2a** are associated with their ability to stabilize the cellular membrane by van-der-Waals interactions with phospholipids of the external layer. This is proved by changes in platelet aggregation induced by arachidonic acid. Arachidonic acid, in contrast to other inducers, activates platelets directly penetrating into the cell, and reduces cyclic adenine monophosphate (cAMP) concentration. Therefore, the loss of this property in the presence of sulfoxide **2a** might be due to the changes in permeability of platelet membranes associated with formation of additional intermolecular interactions between hydrophobic parts of the molecules and the phospholipids of the membrane. Decrease of spontaneous aggregation in the presence of thioterpenoid is associated with increased intracellular cAMP formation due to membrane lipids peroxidation blocking. Integration of the hydrophobic part of sulfoxide **2a** molecule into the cellular membrane induces its stabilization and thereby prevents the utilization of the phosphatidylcholine molecules from the external layer as a formation source of lipid peroxidation products, which in turn will trigger the of platelet aggregation mechanisms. Sulfoxide **2a** also reduced coagulation activity of the plasma: aPTT and prothrombin time became similar to normal values in presence of this compound. These facts indicate suppression of the internal way protrombinaze activation. Platelet activation and significant increase in microvesiculation during storage have been described in several studies. These processes are caused by membrane phospholipids reallocation of and exposure of negatively charged phosphatidylserine both on platelets and on the surface of microvesicles (Lawrie et al., 2009; Lhermusier et al., 2011). The results of our study show that the number of microvesicles changes is an informational marker of platelets activation process (Figure 6). In addition, this reallocation of platelets membrane phospholipids leads to initiation of coagulation hemostasis. Therefore, activation of these processes reduces both the number of functionally active platelets and pro-coagulant reserve of the blood plasma associated with accumulation of thrombogenic particles and activation of the plasma clotting enzyme complexes factors on the surface of membranes. This activity depends on the presence of microvesicles. Coagulation properties of plasma changes in presence of microvesicles from the platelet concentrate: APTT, prothrombin time reduces, and excess thrombin generation leads to acceleration of fibrin clots formation (increased speed of fibrin formation, size, density, and spontaneous clots appearance). Thrombogenic properties of microvesicles are confirmed by the results of their removal from plasma, leading to blood coagulation reduction (increasing fibrin formation initiation time and decreasing of other thrombodynamics indicators, Table 3). The results of our study also demonstrate incomplete stabilization of blood products in case of sodium citrate using. Extracellular calcium ions binding with sodium citrate exclude the possibility of their participation in the activation of platelets and coagulation factors. However, mobilization of intracellular calcium is sufficient for platelet initiating. Intracellular calcium concentration increases after the platelets receptors interaction with inductors—epinephrine, ADP, collagen, and others (Siess, 1989; Shitikova, 2000). The inhibition of receptor activity by sulfoxide **2a** effectively decreased the platelet-derived microvesicles content (Figure 6). Platelet receptors function blocking property of sulfoxide is combined with the anticoagulant effects—aPTT, prothrombinc time and indicators of thrombodynamics reach normal plasma values. The observed hypocoagulative effect of sulfoxide **2a** is induced by reduction of coagulation activity of microvesicles present in solution, because the same changes are caused by their removal from plasma (Table 3). Thrombogenic microvesicles are mainly associated with the presence of phosphatidylserine (Lhermusier et al., 2011) promoting Ca^2+^-dependent binding of its negative charge with vitamin K-dependent coagulation catalytic complexes on the surface of membrane (Cutsforth et al., 1989).

The results of our study revealed that coagulation activity of thioterpenoids is associated with selective inhibition of platelet aggregation and coagulation factors inhibition. Considering the low toxicity, the ability to block spontaneous- and induced-aggregation, sulfur-containing terpene compounds might be considered as the promising agents with perspective use for platelet blood products stabilization, treatment and prevention of thrombophilia.

## Author contributions

LN: General leadership, results discussion, writing the article. SK: Biochemical assays, results discussion. VS: Synthesis. AB: Synthesis, article design. ZA: Biochemical assays. OS: Synthesis. IF: Synthesis. SB: Biochemical assays, statistic analysis. OL: Single-crystal X-ray. VK: NMR spectroscopy. LG: NMR spectroscopy. AK: Molecular dynamics simulations.

### Conflict of interest statement

The authors declare that the research was conducted in the absence of any commercial or financial relationships that could be construed as a potential conflict of interest.

## References

[B1] Abraham M. J. van der Spoel D. Lindahl E. Hess B. The GROMACS Development Team (2014). GROMACS User Manual version 5.0.1. Available online at: www.gromacs.org

[B2] Alfonsov V. A. Bredikhin A. A. Bredikhina Z. A. Eliseenkova R. M. Kataeva O. N. Litvinov I. A. (2008). First examples of the cocrystallization of diastereomers of chiral phosphorus compounds. Struct. Chem. 19, 873–878. 10.1007/s11224-008-9353-4

[B3] Anderson K. M. Afarinkia K. Yu H.-W. Goeta A. E. Steed J. W. (2006). When Z' = 2 Is better than Z' = 1 supramolecular centrosymmetric hydrogen-bonded dimers in chiral systems. Cryst. Growth Des. 6, 2109–2113. 10.1021/cg0603265

[B4] Annunziata R. Cinquini M. Cozzi F. Farina S. (1987). Synthesis of configurationally stable allylic sulphoxides via diastereoselective oxidation. Tetrahedron Lett. 43, 1013–1018. 10.1016/S0040-4020(01)90039-0

[B5] Arai Y. Matsui M. Koizumi T. J. (1991). Powerful dienophiles for asymmetric Diels-Alder reactions:.alpha.-(2-exo-hydroxy-10-bornylsulfinyl)maleimides. Org. Chem. 56, 1983–1985. 10.1021/jo00006a005

[B6] Aversa M. C. Barattucci A. Bonaccorsi P. (2002). Synthesis and Diels-Alder reactivity of sulfinyl homo- and hetero- dienes obtained via enantio-pure sulfenic acids. ARKIVOC 17, 79–98. 10.3998/ark.5550190.0003.b09

[B7] Balick M. Duke J. Kaptchuk T. Mc Coleb R. Pavek R. Pellin C. (1994). Herbal Medicine. Washington, DC: National Institutes of Health.

[B8] Berendsen H. J. C. Postma J. P. M. Gunsteren W. F. Hermans J. (1981). Interaction models for water in relation to protein hydration, in Intermolecular Forces, ed Pullman B. (Dordrecht: Reidel), 331–342.

[B9] Binns M. R. Goodridge R. J. Haynes R. K. (1985). Preparation of stable, camphor-derived, optically active allylic sulfoxides. Tetrahedron Lett. 26, 6381–6384. 10.1016/S0040-4039(01)84604-9

[B10] Born G. (1962). Aggregation of blood platelets by adenosine diphosphate and its reversal. Nature 194, 927–929. 10.1038/194927b0 13871375

[B11] Bruce C. D. Berkowitz M. L. Perera L. Forbes M. D. E. (2002). Molecular dynamics simulation of sodium dodecyl sulfate micelle in water: micellar structural characteristics and counterion distribution. J. Phys. Chem. 106, 3788–3793. 10.1021/jp013616z

[B12] Caillon L. Lequin O. Khemtémourian L. (2013). Evaluation of membrane models and their composition for islet amyloidpolypeptide-membrane aggregation. Biochim. Biophys. Acta 1828, 2091–2098. 10.1016/j.bbamem.2013.05.014 23707907

[B13] Carreno M. C. Hernandez-Torres G. Ribagorda M. Urbano A. (2009). Enantiopure sulfoxides: recent applications in asymmetric synthesis. Chem. Commun. 41, 6129–6144. 10.1039/b908043k 19826652

[B14] Cutsforth G. A. Whitaker R. N. Hermans J. Lentz B. R. (1989). A new model to describe extrinsic protein binding to phospholipid membranes of varying composition: application to human coagulation proteins. Biochemistry 28, 7453–7461. 10.1021/bi00444a045 2819080

[B15] Demakova M. Y. Sudarikov D. V. Rubtsova S. A. Popov A. V. Frolova L. L. Slepukhin P. A. (2012a). Synthesis and asymmetric oxidation of caranylsulfanylimidazoles. Helv. Chim. Acta 5, 940–950. 10.1002/hlca.201100484

[B16] Demakova M. Y. Sudarikov D. V. Rubtsova S. A. Slepukhin P. A. Kutchin A. V. (2012b). Reaction of 1-methyl-2-terpenylsulfanylimidazoles with chlorine dioxide. Russ. J. Org. Chem. 1, 113–118. 10.1134/S1070428012010186

[B17] Eschler B. Haynes R. K. Kremmydas S. J. (1988). A simple route to (R)-(+)-4-t-butoxycyclopent-2-enone. Chem. Soc. Chem. Commun. 137–138. 10.1039/c39880000137

[B18] Finegold J. A. Asaria P. Francis D. P. (2013). Mortality from ischemic heart disease by country, region, and age: statistics from World Health Organisation and United Nations. Int. J. Cardiol. 168, 934–945. 10.1016/j.ijcard.2012.10.046 23218570 PMC3819990

[B19] Fucke K. Qureshi N. Yufit D. S. Howard J. A. K. Steed J. W. (2010). Hydrogen bonding is not everything: extensive polymorphism in a system with conserved hydrogen bonded synthons. Cryst. Growth Des. 10, 880–886. 10.1021/cg901224f

[B20] Galiullina L. F. Blokhin D. S. Aganov A. V. Klochkov V. V. (2012). Investigation of cholesterol+model of biological membrane complex by NMR spectroscopy. MRSej. 14:12204 Available online at: https://www.scopus.com/inward/record.uri?eid=2-s2.0-84871625991&partnerID=40&md5=54fa000ff2c16e23dbaa04259fa29fff

[B21] Galiullina L. F. Rakhmatullin I. Z. Klochkova E. A. Aganov A. V. Klochkov V. V. (2015). Structure of pravastatin and its complex with sodium dodecyl sulfate micelles studied by NMR spectroscopy. Magn. Reson. Chem. 53, 110–114. 10.1002/mrc.4146 25264019

[B22] Gasparyan A. Yu Watson T. Lip G. Y. H. (2008). The role of aspirin in cardiovascular prevention: implications of aspirin resistance. J. Am. Coll. Cardiol. 51, 1829–1843. 10.1016/j.jacc.2007.11.080 18466797

[B23] Gavrilov V. V. Startseva V. A. Nikitina L. E. Lodochnikova O. A. Gnezdilov O. I. Lisovskaya S. A. (2010). Synthesis and antifungal activity of sulfides, sulfoxides, and sulfones based on (1S)-(–)-β-pinene. Pharm. Chem. J. 44, 126–129. 10.1007/s11094-010-0413-x

[B24] Grant D. J. W. (1999). Theory and origin of polymorphism, in Polymorphism in Pharmaceutical Solids, ed Brittain H. G. (New York, NY: Marcel Dekker Inc), 1–34.

[B25] Henry G. D. Sykes B. D. (1994). Methods to study membrane protein structure in solution. Meth. Enzymol. 239, 515–535. 10.1016/S0076-6879(94)39020-7 7830597

[B26] Humphrey W. Dalke A. Klaus S. (1996). VMD - Visual Molecular Dynamics. J. Mol. Graph. 14, 33–38. 10.1016/0263-7855(96)00018-5 8744570

[B27] Ishmuratov G. Y. Yakovleva M. P. Tukhvatshin V. S. Talipov R. F. Nikitina L. E. Artemova N. P. (2014). Sulfurcontaining derivatives of mono- and bicyclic natural monoterpenoids. Chem. Nat. Compd. 50, 22–47. 10.1007/s10600-014-0862-7

[B28] Iversen L. V. Ostergaard O. Nielsen C. T. Jacobsen S. Heegaard N. H. (2013). A heparin-based method for flow cytometric analysis of microparticles directly from platelet-poor plasma in calcium containing buffer. J. Immunol. Methods 388, 49–59. 10.1016/j.jim.2012.12.001 23246793

[B29] Kiselev S. V. Nikitina L. E. Startseva V. A. Artemova N. P. Bodrov A. V. Boichuk S. V. (2017). Hemocoagulation activity of sulfur-containing pinane-type terpenoids. Pharm. Chem. J. 51, 78–82. 10.1007/s11094-017-1611-6

[B30] Komatsu N. Hashizume M. Sugita T. Uemura S. (1993). Catalytic Asymmetric oxidation of sulfides with tret-Butyl hydroperoxide using binaphthol as a chiral auxiliary. J. Org. Chem. 58, 4529–4533. 10.1021/jo00069a009

[B31] Krudysz-Amblo J. Mann K. G. Butenas S. (2015). Tissue factor structure and coagulation, in Thrombosis and Inflammation in Acute Coronary Syndromes, eds Ercan E. Ece G. (Sharjah: Bentham Science Publishers), 23–57.

[B32] Lawrie A. S. Albanyan A. Cardigan R. A. Mackie I. J. Harrison P. (2009). Microparticle sizng by dynamic light scattering in freshfrozen plasma. Vox Sang. 96, 206–212. 10.1111/j.1423-0410.2008.01151.x 19175566

[B33] Lhermusier T. Chap H. Payrastre B. (2011). Platelet membrane phospholipid asymmetry: from the characterization of a scramblase activity to the indentification of an essential protein mutated in Scott syndrome. J. Thromb. Haemost. 9, 1883–1891. 10.1111/j.1538-7836.2011.04478.x 21958383

[B34] Lodochnikova O. A. Krivolapov D. B. Startseva V. A. Nikitina L. E. Bodrov A. V. Artemova N. P. (2015). S = O…S = O interactions as a driving force for low-temperature conformational rearrangement of stable H-bonding {S(O)-CH_2_-CH_2_-OH∙∙∙}_2_ synthon in two modifications of diastereomeric pinanyl sulfoxides co-crystal. Phosphorus Sulfur Silicon Relat. Elem. 190, 2222–2231. 10.1080/10426507.2015.1072185

[B35] Lubecka E. Kwiatowska A. Ciarkowski J. Sikorska E. (2010). NMR studies of new arginine vasopressin analogs modified with α-2-indanylglycine enantiomers at position 2 bound to sodium dodecyl sulfate micelles. Biophys. Chem. 151, 139–148. 10.1016/j.bpc.2010.06.002 20598431

[B36] MacKerell A. D. Jr. (1995). Molecular dynamics simulation analysis of a sodium dodecyl sulfate micelle in aqueous solution: decreased fluidity of the micelle hydrocarbon interior. J. Phys. Chem. 99, 1846–1855. 10.1021/j100007a011

[B37] Malde A. K. Zuo L. Breeze M. Stroet M. Poger D. Nair C. P.. (2011). An automated force field topology builder (ATB) and repository: version 1.0. J. Chem. Theory Comput. 7, 4026–4037. 10.1021/ct200196m 26598349

[B38] Nikitina L. E. Akulina I. V. Garaev R. S. Artemova N. P. Dorofeeva L. Y. Startseva V. A. (2012a). Synthesis and anti-inflammatory and antipyretic activity of 2-(1'-hydroxy-4'-isopropenyl-1'-methylcyclohexyl-2'-thio)-methylethanoate. Pharm. Chem. J. 46, 20–22. 10.1007/s11094-012-0727-y

[B39] Nikitina L. E. Artemova N. P. Startseva V. A. (2011). Natural and Thiomodified Monoterpenoids. Saarbrucken: LAP LAMBERT.

[B40] Nikitina L. E. Artemova N. P. Startseva V. A. Fedyunina I. V. Klochkov V. V. (2017a). Biological activity of S-containing monoterpenoids. Chem. Nat. Compd. 5, 811–819. 10.1007/s10600-017-2131-z

[B41] Nikitina L. E. Dieva S. A. Plemenkov V. V. Lodochnikova O. A. Gubaidullin A. T. Kataeva O. N. (2001). 7,7-Dimethyl-2,10-epoxybicyclo[3.1.1]heptane. Synthesis, structure, and products of epoxide ring cleavage. Russ. J. Gen. Chem. 8, 1233–1237. 10.1023/A:1013251021230

[B42] Nikitina L. E. Kiselev S. V. Bodrov A. V. Startseva V. A. Artemova N. P. Klochkov V. V. (2017b). Development of approaches to the study of the interaction of biologically active thioterpenoids with model membranes. BioNanoScience 7, 600–607. 10.1007/s12668-017-0432-0

[B43] Nikitina L. E. Lodochnikova O. A. Startseva V. A. Bodrov A. V. Artemova N. P. Klimovitskii A. E. (2017c). Extraordinary behavior of β-hydroxy sulfoxides and sulfone of pinane series. Phosphorus Sulfur Silicon Relat. Elem. 192, 187–191. 10.1080/10426507.2016.1255619

[B44] Nikitina L. E. Startseva V. A. Artemova N. P. Dorofeeva L. Yu Kuznetsov I. V. Lisovskaya S. A. (2012b). Synthesis and antifungal activity of the carane series. Pharm. Chem. J. 45, 664–667. 10.1007/s11094-012-0699-y

[B45] Nikitina L. E. Startseva V. A. Dieva S. A. Vakulenko I. A. Shamov G. A. (2006). Reaction of β-pinene and thiols in the presence of Lewis acids. Chem. Nat. Compd. 2, 178–181. 10.1007/s10600-006-0072-z

[B46] Nikitina L. E. Startseva V. A. Dorofeeva L. Y. Artemova N. P. Kuznetsov I. V. Lisovskaya S. A. (2010). Antifungal activity of bicyclic monoterpenoids and terpenesulfides. Chem. Nat. Compd. 46, 28–32. 10.1007/s10600-010-9517-5

[B47] Nikitina L. E. Startseva V. A. Vakulenko I. A. Khismatulina I. M. Lisovskaya S. A. Glushko N. P. (2009). Synthesis and antifungal activity of compounds of the pinane series. Pharm. Chem. J. 43, 251–254. 10.1007/s11094-009-0282-3

[B48] Niks M. Otto M. (1990). Towards an optimized MTT-assay. J. Immunol. Meth. 130, 149–151. 10.1016/0022-1759(90)90309-J 2358686

[B49] Pyne S. G. Bloem P. Griffith R. (1989). Conjugate addition of amines to (Rs)-10-isobornyl vinyl sulfoxides. Tetrahedron Lett. 45, 7013–7022. 10.1016/S0040-4020(01)89169-9

[B50] Rakhmatullin I. Z. Galiullina L. F. Klochkova E. A. Latfullin I. A. Aganov A. V. Klochkov V. V. (2016). Structural studies of pravastatin and simvastatin and their complexes with SDS micelles by NMR spectroscopy. J. Mol. Struct. 1105, 25–29. 10.1016/j.molstruc.2015.10.059

[B51] Santos M. R. V. Moreira F. V. Fraga B. P. De Sousa D. P. Bonjardim L. R. Quintans-Junior L. J. (2011). Cardiovascular effects of monoterpenes: a review. Rev. Bras. Farmacogn. 21, 764–771. 10.1590/S0102-695X2011005000119

[B52] Shitikova A. S. (2000). Thrombocytic Hemostasis. Saint Petersburg: Publishing house of Saint Petersburg State Medical University.

[B53] Siess W. (1989). Molecular mechanism of platelet activation. Physiol. Rev. 69, 58–78. 10.1152/physrev.1989.69.1.58 2536184

[B54] Startseva V. A. Nikitina L. E. Lodochnikova O. A. Klimovitskii A. E. Aref'ev A. V. Artemova N. P. (2014). Study of “Racemic Compound-Like” behavior of diastereomeric mixture of pinanyl sulfoxides by X-ray diffraction, IR spectroscopy, and DFT calculations. Phosphorus Sulfur Silicon Relat. Elem. 189, 615–629. 10.1080/10426507.2013.843003

[B55] Usachev K. S. Filippov A. V. Filippova E. A. Antzutkin O. N. Klochkov V. V. (2013). Solution structures of Altzheimers amyloid Aβ13-23 peptide: NMR studies in solution and in SDS. J. Mol. Struct. 1049, 436–440. 10.1016/j.molstruc.2013.06.043

[B56] Vargas-Díaz M. E. Lagunas-Rivera S. Joseph-Nathan P. Tamariz J. Zepeda L. G. (2005). Efficient and highly diastereoselective preparation of a myrtenal derived bis-sulfoxide and its preliminary evaluation as chiral acyl donor. Tetrahedron Lett. 46, 3297–3300. 10.1016/j.tetlet.2005.03.104

[B57] Wojaczynska E. Wojaczynski J. (2010). Enantioselective synthesis of sulfoxides: 2000-2009. Chem. Rev. 110, 4303–4356. 10.1021/cr900147h 20415478

[B58] Yang T. K. Chen R.-Y. Lee D.-S. (1994). Application of new camphor-derived mercapto chiral auxiliaries to the synthesis of optically active primary amines. J. Org. Chem. 59, 914–921. 10.1021/jo00083a037

